# Benchmarking neuromorphic vision: lessons learnt from computer vision

**DOI:** 10.3389/fnins.2015.00374

**Published:** 2015-10-13

**Authors:** Cheston Tan, Stephane Lallee, Garrick Orchard

**Affiliations:** ^1^Agency for Science, Technology, and Research (A^*^STAR), Institute for Infocomm ResearchSingapore, Singapore; ^2^Singapore Institute for Neurotechnology (SINAPSE), National University of SingaporeSingapore, Singapore; ^3^Temasek Labs, National University of SingaporeSingapore, Singapore

**Keywords:** neuromorphic vision, computer vision, benchmarking, datasets, sensory processing

## Abstract

Neuromorphic Vision sensors have improved greatly since the first silicon retina was presented almost three decades ago. They have recently matured to the point where they are commercially available and can be operated by laymen. However, despite improved availability of sensors, there remains a lack of good datasets, while algorithms for processing spike-based visual data are still in their infancy. On the other hand, frame-based computer vision algorithms are far more mature, thanks in part to widely accepted datasets which allow direct comparison between algorithms and encourage competition. We are presented with a unique opportunity to shape the development of Neuromorphic Vision benchmarks and challenges by leveraging what has been learnt from the use of datasets in frame-based computer vision. Taking advantage of this opportunity, in this paper we review the role that benchmarks and challenges have played in the advancement of frame-based computer vision, and suggest guidelines for the creation of Neuromorphic Vision benchmarks and challenges. We also discuss the unique challenges faced when benchmarking Neuromorphic Vision algorithms, particularly when attempting to provide direct comparison with frame-based computer vision.

## 1. Introduction

Benchmarking using widely accepted datasets is important for algorithm development. Such benchmarking allows quantitative performance evaluation and comparison between algorithms, promoting competition and providing developers with tangible state-of-the-art targets to beat. Computer Vision (CV) is an obvious example where open access to good datasets has been integral in rapid development and maturation of the field (Kotsiantis et al., 2006).

We use the term “Computer Vision” (CV) to denote the conventional approach to visual sensing, which begins with acquisition of images (photographs), or sequences of images (video). Each image is a regular grid of pixels, each pixel having an intensity or color value. Such images are a widely accepted, and largely unquestioned first step in visual sensing.

However, the much younger field of Neuromorphic Vision (NV) takes a radically different approach, doing away with images completely. The term “Neuromorphic Vision” (NV) denotes approaches which rely on custom designed bio-inspired vision sensors which capture data in a non-frame-based manner. The most mature and common of these sensors are the event-based asynchronous temporal contrast vision sensors. Other NV sensors have not yet reached a level of maturity where they can be used to reliably capture datasets. Nevertheless, the opinions and guidelines stated in this paper extend to benchmarks and datasets for other NV sensors once they do reach such a level of maturity.

**Figure 1:**
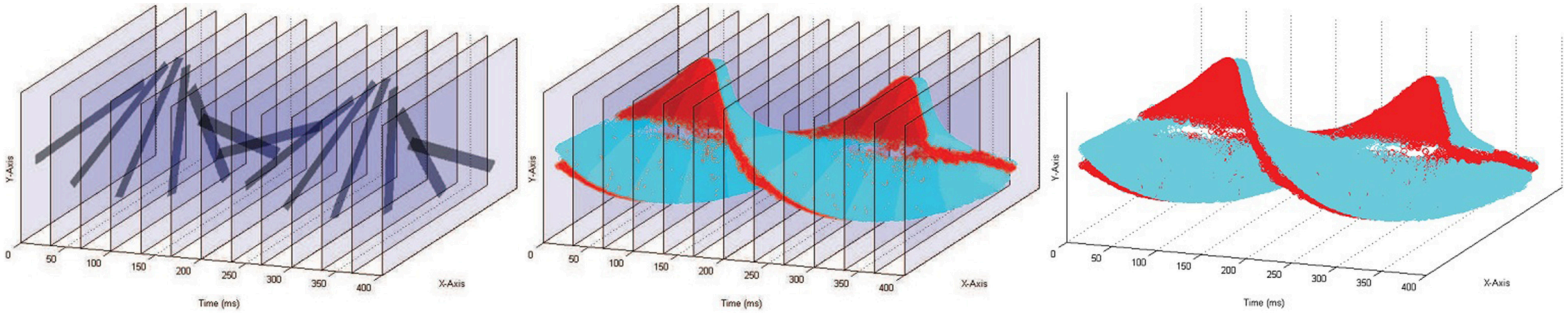
**Comparison of data formats between CV and temporal contrast NV for a rotating black bar stimulus**. **Left:** simulated 30fps recording of a black spinning bar. Every pixel's intensity value is captured at constant time intervals. The location of the bar in each frame can be seen, but the location of the bar between frames must be inferred. **Right:** Actual recording of a rotating bar captured with a NV sensor. Blue and red points indicate *on* (increasing intensity) and *off* (decreasing intensity) events respectively. Each pixel immediately outputs data when it detects a change in intensity. **Middle:** Superposition of the NV and CV data. The NV sensor captures intensity changes occurring between the CV frames (blue and red data points), but does not recapture redundant background pixel intensities (indicated by transparent frame regions) as is done in the CV format. A video accompanying this figure is available online^1^.

For temporal contrast NV sensors (hereafter referred to simply as “NV sensors”), each pixel generates an event whenever its change in log-intensity over time exceeds a programmable threshold. In these sensors, any pixel can generate an event at any time, thereby accurately recording when and where scene changes occur. Figure 1 shows how CV and temporal contrast NV data formats differ. CV data arrives from every pixel at regular time intervals (frame intervals), whereas NV data is received only when and where temporal pixel changes occur (Posch et al., 2014).

The potential for NV to benefit from datasets similar in quality to those of CV is well recognized, in part leading to the launch of this special topic. However, dataset creation for NV poses unique challenges. In this article we discuss these challenges (Section 2) and assess the current state of NV datasets (Section 3) before reviewing the role datasets have played in CV (Section 4), identifying valuable lessons (Section 5) and how NV can benefit from these lessons (Section 6).

## 2. Challenges in benchmarking neuromorphic vision

The main difficulty in benchmarking NV arises because NV data differs significantly from CV data (Figure 1) and no accurate method for converting between data formats exists. NV video is captured with temporal resolution of a few microseconds (Posch et al., 2014), while CV video is captured with temporal resolution of tens of milliseconds. Thus, high temporal frequencies are lost during frame-based CV sampling and cannot be reconstructed to simulate NV.

Converting video from NV to CV is also problematic. NV detects changes in intensity, and attempts to estimate absolute intensity (as provided by CV sensors) from these changes have thus far proved largely unsuccessful. However, some recent works (Kim, 2014) show that it is possible in limited cases (under sensor rotation but not translation).

For static images, direct comparison between NV and CV proves even more difficult. A static NV sensor viewing a static scene will observe no changes, and therefore no data will be captured (except for some noise). To capture data, the NV sensor must either be viewing a dynamic scene, or the sensor must be moving. In biology, this problem is avoided by ensuring that the sensor (eye or retina) is always moving (Martinez-Conde et al., 2004).

Even when fixating on a point, fixational eye movements (*drifts* and *microsacades*) continue. Originally, *drift* was thought of as an error in the eye's attempt to fixate, and *microsaccades* were thought to be corrections for this error, but more recent research suggests that fixational eye movements are important for perception (Engbert, 2006).

Motion of a NV sensor can similarly be used to generate data while viewing a static image, providing a means to convert static image (but not video) datasets from CV to NV. This method of converting datsets allows comparison between CV and NV, and allows NV datasets to be created from existing CV datasets, saving the considerable time required to collect and annotate data. This conversion process is presented in a separate paper in this special topic.

Although there are many lucrative applications involving recognizing objects in static images, this is not the primary task that evolution has optimized biological vision to solve. Static organisms have little use for vision (plants sense light, but cannot “see”). Rather, biological vision has evolved on mobile embodied agents existing in a dynamic world. The bio-inspired NV approach is therefore better suited to similar scenarios: sensing for mobile agents. Such applications require processing video, not static images.

Directly comparing CV and NV video results requires simultaneous recording from real world scenes in both formats. Simultaneous recording prohibits using existing CV videos for dataset creation, which is how CV datasets are typically created (Socher, 2009). Recording one's own video is far more time consuming, although recent NV sensors (Posch et al., 2011; Berner et al., 2013) do allow simultaneous grayscale and change detection recording.

Once such a dataset is created, there remains the problem of ensuring it is recognized and adopted by both the CV and NV communities, such that competitive comparisons across communities can occur. Although not all datasets are intended to enable comparison between CV and NV, a lack of available NV recordings still prohibits the gathering of pre-existing data as a means of dataset creation.

## 3. Current state of neuromorphic vision datasets

Although silicon retinae first appeared over 25 years ago (Mead, 1989), only in the last decade have NV sensors entered the commercial market. Despite advances in sensor design and increasing interest in spike-based computation for vision, a lack of good NV datasets remains. Most NV datasets created thus far can be assigned to one of three categories.

**Figure 2:**
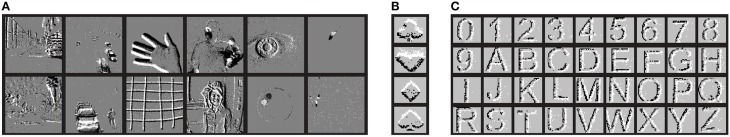
**Examples of NV data**. **(A)** Publicly available unannotated sequences. From left to right (both rows) dashcam recordings, static surveillance of street scenes, test stimuli, juggling and first person walking video, high speed eye tracking and dot tracking, and rat and fly behavioral recordings. **(B)** A small annotated card pip dataset consisting of 10 examples of each of the 4 card pips (Pérez-Carrasco et al., 2013). **(C)** Another small annotated dataset, consisting of 2 examples of each of the 36 characters (Orchard et al., 2015).

The first category consists of unannotated recordings which provide interested researchers with access to NV data. Examples include data provided with the opensource jAER software^2^, which consists of recordings under a various scenarios (Figure 2A).

The second category consists of small annotated datasets, each created to showcase performance of a particular algorithm (Figures 2B,C; Pérez-Carrasco et al., 2013; Orchard et al., 2015). These datasets are created out of necessity in quantifying algorithm performance because no prior datasets are adequate. These datasets are small and near 100% accuracy is soon achieved using subsequent algorithm improvements, after which a new dataset must once again be recorded.

The third category consists of large annotated datasets created specifically for the purpose of providing the NV community with good datasets. These datasets are the most valuable, but also the fewest in number. The best known is the MNIST DVS dataset^3^ created from the CV MNIST dataset^4^ using a static sensor observing a CV video generated from static images. It therefore suffers from the temporal frequency problem discussed in Section 2.

Not all researchers investigating spike-based computation for vision intend for their algorithms to be used with NV sensors. Many works instead make points about biological vision (Masquelier and Thorpe, 2007), the potential of spike-based computation (Andreopoulos et al., 2015), or how spike-based vision can leverage existing methods from CV. In these works, the spiking input artificially simulated from static images is not necessarily intended to mimic a NV sensor.

There is a glaring lack of large annotated NV datasets, but given the rapidly growing interest in NV, it is inevitable that such datasets will appear soon, and these datasets will heavily influence NV research for the near future. These datasets should therefore be created with care and deliberation. Recognizing this unique opportunity to shape NV, and the potential to benefit from hindsight in the more mature field of CV, we turn our attention to analyzing the roles datasets have played in the development of CV.

## 4. Brief history and evolution of frame-based vision datasets

The impact of datasets on CV research (particularly object recognition) is perhaps best summarized by Torralba and Efros (2011):

“They have been the chief reason for the considerable progress in the field, not just as a source of large amounts of training data, but also as a means of measuring and comparing performance of competing algorithms. At the same time, datasets have often been blamed for narrowing the focus of object recognition research, reducing it to a single benchmark performance number.”

In this section we focus on datasets for object recognition, primarily because the impact has been greatest for this area of CV.

The 1990s saw the start of the era of CV that utilized techniques from machine learning and statistics to learn from labeled examples. The MNIST dataset of handwritten digits contained a total of 70,000 examples of the 10 digits. The Caltech-5 dataset^5^ had over 3000 images from 5 categories. However, these datasets had limited variability. For example, the digits in MNIST had uniform backgrounds, while the Caltech-5 objects did not have pose variations (e.g., all motorbikes are viewed from the side).

The next generation of datasets (early-to-mid 2000s) were characterized by a trade-off between dataset size and real-world representativeness. Caltech-101^6^ had almost 10,000 images from 101 categories, featured complex backgrounds, and had variations of object instances within their categories. However, the dataset was criticized as not being representative of natural images because the objects were all centered in the image. Partly in reaction to this criticism, the PASCAL VOC 2005 Challenge dataset^7^ made a point of including as much variability in object position, scale, view, etc. as possible, but was smaller in size (3500 images from only 4 categories).

In the late 2000s, massive datasets were created as a result of advances in dataset collection techniques, as well as concerted collection efforts once the importance of large, well-designed datasets was recognized. The Tiny Images dataset^8^ had almost 80 million images related to 53,464 nouns from the WordNet ontology (Miller, 1995), although initially only a fraction of the images were annotated. The ImageNet dataset^9^, which had 1.4 million images from 1000 categories for its 2010 Challenge, also used WordNet. Meanwhile, the PASCAL VOC Challenge scaled up its efforts, while maintaining its focus on image variability rather than a large number of categories. This trend of larger and larger datasets continues up to the present time. The Yahoo Flickr Creative Commons dataset^10^ is currently the largest with 100 million images. Over 15 years, datasets have grown 10,000 times as large, almost doubling every year.

## 5. Lessons learnt from computer vision datasets

### 5.1. Technical lessons

The biggest technical lesson is that CV datasets should be as representative of the “real visual world” as possible (Torralba and Efros, 2011). This could be the physical world experienced by our eyes and cameras, or it could also be the world of internet images. If datasets deviate from the characteristics of the world(s) they are supposed to represent, then good dataset performance is unlikely to translate into good real world performance.

In some ways, datasets have evolved along the path of increasingly realistic representation of the visual world both quantitatively (number of categories and images) and qualitatively (more natural, variable, and noisy). For example, the PASCAL VOC datasets were designed to go beyond Caltech101 images which primarily had a single large, unoccluded, centered object. Caltech101 itself was created to go beyond the small number of categories in prior datasets. Moreover, the trend of increasing variability in the data is also present on the algorithm side. An increasingly common technique (Van Dyk and Meng, 2001; Krizhevsky et al., 2012) is to augment a dataset by applying transformations to it (e.g., noise and color channel manipulations) in an attempt to increase the input space explored. Similar approaches can be considered for NV, such as randomly varying an event's pixel location or timestamp.

Related to the above is the lesson of being aware of dataset bias, which includes selection, capture, labeling, and negative set bias (Torralba and Efros, 2011). One way to reduce effects of such biases—though not eliminate it—is to include cross-dataset generalization performance as a performance metric. Additionally, the flaws in the data can be explicitly considered in order to tune algorithms during the training phase, to overcome bias effects (e.g., dealing with unbalanced datasets Kotsiantis et al., 2006).

### 5.2. Meta lessons

In this section, we review some of the “meta lessons” learnt (those beyond the content of the datasets themselves). The first lesson is that collecting large bias-minimized datasets requires giving thorough thought to collection and annotation techniques. Indeed, the Big Data revolution in object recognition would not have come about if not for automated techniques for amassing labeled image collections. Furthermore, such efforts must be recognized by the community as important, rather than secondary to algorithm development (Russell et al., 2005; Sapp et al., 2008).

Another lesson is the importance of avoiding “creeping over-fitting” which arises when datasets are treated as a world in their own right, and “too much value [is given] to winning a particular challenge” (Torralba and Efros, 2011), regardless of whether there is actually any statistical significance among various competing algorithms. At the very least, statistical significance should be taken into account, as is done in PASCAL VOC (Everingham et al., 2014). However, statistically significantly higher accuracy on a dataset may still be a result of overfitting to the dataset, which is not desirable if the algorithm is intended for real-world application. It may not be fundamentally correct to treat dataset performance as a competition among individual algorithms, but perhaps rather as verification that an algorithm possesses some minimal generalization performance above baseline algorithms. This relates to a growing issue with dataset-based benchmarking: as the research becomes overly focused on increasing dataset performance, the original purposes and constraints may become forgotten.

Another lesson is the importance of rigorous evaluation protocols that explicitly specify training and testing conditions, so that researchers can unambiguously do “apples-to-apples” comparisons. This does not mean there can only be one numerical metric. While a single metric has advantages as a simple direct objective measure, a single number can rarely capture everything. Most recent papers also include qualitative descriptions, e.g., common error types, accompanied by a few typical examples. Furthermore, a performance metric on its own is not very informative. Performance may be significantly above chance, but exactly how good is it? A good pool of baseline methods (for example, a standard reference collection of formerly state-of-the-art methods) is important for gauging how far the field has progressed.

Linked to the above point about good baseline methods, a final lesson is the importance of a culture of ensuring that results are reproducible. There is no better way than to release source code (instead of just having equations or pseudo-code, or even pre-compiled executables). In CV, such efforts have been rewarded by higher citation rates; all other things being equal, authors will naturally use (and therefore cite) as comparison previous work that comes with publicly-available code that can be re-run easily. Instead of re-implementing from scratch these methods, researchers can concentrate on developing better algorithms, which in turn will be used for comparison. This virtuous cycle speeds up research progress for everyone.

## 6. Recommendations for neuromorphic vision

The primate visual system is sometimes simplified down to a sustained and transient pathway, which respond to static and changing stimuli respectively. Although different, each pathway has its own unique strengths and the two operate in a complementary manner. Similarly, although CV and NV differ significantly, the two should be seen as complementary approaches, with each having its own strengths. It is tempting to quantify the value of NV using existing CV tasks and metrics, but these have been designed to quantify the value of CV because it has largely shaped the field of artificial visual sensing thus far. NV researchers should instead focus on metrics which show the strengths of NV, while remaining honest about the impressive capabilities of CV.

NV datasets should be created to aid development of algorithms and metrics in areas where NV shows the most promise. These areas include retinal prostheses and visual sensing for mobile agents, both of which capture data from a first-person viewpoint. Also, sensing from mobile platforms typically imposes size, weight, and power consumption constraints, which performance metrics should take into account in addition to accuracy, as is done in the DARPA's Stanford Tower NeoVision2 dataset^11^. Furthermore, sensing from a mobile platform opens the possibility of active perception (moving the sensor in a manner that maximizes the visual information acquired) which is often overlooked in bio-inspired visual sensing. These are the areas in which NV datasets should be created.

As learnt from CV, datasets should be representative of the final application. For the examples above, this means recording NV video using moving rather than static sensors. When recording, one should attempt to capture the variability expected in the final application and avoid using simulators that do not accurately recreate the noise variability introduced by actual NV sensors. It will also be beneficial to NV to have comparisons to CV, which would mean creating simultaneously recorded datasets of high enough quality to attract CV researchers.

We recognize that recording and annotating NV data, particularly video, is a difficult and time consuming task (Section 2). However, the value of good datasets is well-documented in CV. Given the degree to which NV stands to benefit from the introduction of similarly good datasets, the present lack of such datasets should be addressed. Dataset creation should not be treated as a secondary task only to showcase the performance of a single algorithm. Rather, similarly to CV, dataset creation should be prioritized and recognized as a task of utmost importance to the field for which time and funding should be specifically allocated.

## Funding

This work was supported by A^*^STAR JCO Grant #1335h00098.

### Conflict of interest statement

The authors declare that the research was conducted in the absence of any commercial or financial relationships that could be construed as a potential conflict of interest.

## Footnotes

^1^https://youtu.be/up_US4EoD0M.

^2^http://sourceforge.net/projects/jaer/.

^3^www.imse-cnm.csic.es/caviar/MNISTDVS.html.

^4^http://yann.lecun.com/exdb/mnist.

^5^http://www.vision.caltech.edu/html-files/archive.html.

^6^http://www.vision.caltech.edu/Image_Datasets/Caltech101.

^7^http://host.robots.ox.ac.uk/pascal/VOC/.

^8^http://groups.csail.mit.edu/vision/TinyImages/.

^9^http://www.image-net.org.

^10^http://webscope.sandbox.yahoo.com/catalog.php?datatype=i&did=67.

^11^http://ilab.usc.edu/neo2/dataset/.
